# *Lagenophora* (Asteraceae, Astereae) in New Caledonia

**DOI:** 10.3897/phytokeys.177.63116

**Published:** 2021-05-13

**Authors:** Guillaume Lannuzel, Gildas Gâteblé, Anthony R.Bean, Jian Wang

**Affiliations:** 1 Institut Agronomique néo-Calédonien, Equipe ARBOREAL, BP 711, 98810, Mont-Dore, New Caledonia Institut Agronomique néo-Calédonien Mont-Dore New Caledonia (Fr); 2 Endemia, Plant Red List Authority, BP 4682, 98847, Nouméa, New Caledonia Endemia, Plant Red List Authority Noum&eacute;a New Caledonia (Fr); 3 Queensland Herbarium, Department of Environment and Science, Brisbane Botanic Gardens, Mt Coot-tha Road, Toowong, Queensland 4066, Australia Queensland Herbarium, Department of Environment and Science Toowong Australia

**Keywords:** Asteroideae, Compositae, identification key, *Lagenophora sinuosa*, *Lagenophora sublyrata*, Lagenophorinae, New Caledonia flora, new species

## Abstract

The genus *Lagenophora* Cass. is taxonomically revised for New Caledonia with two species recognised. *Lagenophora sinuosa* Lannuzel, Gâteblé & Jian Wang ter, **sp. nov.** is endemic to New Caledonia and the other, *L. sublyrata* (Cass.) A.R.Bean & Jian Wang ter occurs there and in many other countries from the region. Both are fully described and illustrated. An identification key is provided, as are notes on the distribution (including maps), habitat, phenology and conservation status. The generic placement of the new species is also discussed.

## Introduction

*Lagenophora* Cass. belongs to subfamily Asteroideae (Cass.) Lindl., tribe Astereae Cass. and subtribe Lagenophorinae G.L.Nesom and is found mainly in the southern hemisphere (Nesom and Robinson 2007). Presently, there are 12 species in Australia (Wang and Bean 2019), nine species in New Zealand (Breitwieser et al. 2012), three species in southern South America (Cabrera 1966; Pruski 2017), two in New Guinea (Wang and Bean 2020) and two in New Caledonia (Munzinger et al. 2020+). The genus also extends to Indonesia, and as far as Sri Lanka and southern Japan (Walker 1976; Wang and Bean 2019, 2020). The genus is considered non-monophyletic as species of *Solenogyne* Cass. are nested between the two main clades of *Lagenophora* retrieved by Sancho et al. (2015).

The genus was apparently first recorded in a publication for New Caledonia by Schlechter (1906) who identified his own specimen (*Schlechter 14804*) as *L. billardierei* Cass. An earlier specimen (*Pancher 473*, P03292495) collected during the 1860s was, however, also identified as a *Lagenophora* by its collector. Other early specimens from this period by Balansa, Baudouin, Deplanche, Germain and Vieillard were either unidentified at the genus level or identified as *Strongylosperma reptans* Benth. [now synonymous to *Leptinella reptans* (Benth.) D.G.Lloyd & C.J.Webb]. In his catalogue of the plants of New Caledonia, Guillaumin (1911: 177) identified most of these early specimens as *L. billardierei*. Later, Moore (1921: 345) also identified some Compton specimens as *L. billardierei* and described a new species under *L. neocaledonica* S.Moore. Guillaumin maintained the two aforementioned names under *Lagenophora* in his revision of New Caledonian Asteraceae (Guillaumin 1937) and in his flora of New Caledonia (Guillaumin 1948) while the name *L. neocaledonica* was excluded from *Lagenophora* by Cabrera (1966: 307) and then placed in the synonymy of *Pytinicarpa sarasinii* (Däniker) G.L.Nesom by Nesom (2001). In addition, Cabrera (1966) listed two species of *Lagenophora* for New Caledonia viz. *L. lanata* A.Cunn. for the Schlechter specimen and *L. gracilis* Steetz for all the other specimens, *L. billardierei* being synonymous with *L. stipitata* (Labill.) Druce, an Australian taxon not present in New Caledonia. Wang and Bean (2019) put *L. lanata* into synonymy under *L. sublyrata* (Cass.) A.R.Bean & Jian Wang ter and narrowed the definition of *L. gracilis* as a Western Australian endemic species. After searches and studies over years, we recognise two species for New Caledonia – one is the indigenous *L. sublyrata*, the other one being a new endemic species described here.

## Materials and methods

This revision is based on morphological examination of *Lagenophora* material from the following herbaria: BRI, CANB, L, MEL, NOU, NSW and P (acronyms following Thiers 2020+). Images of type specimens held at BM, FI, G, HAL, K, M, NY, P and W have also been examined. An exclamation mark (!) is used for the specimens physically seen while “image!” is used for specimens seen only by means of digitized images. In January 2017, a whole shipment of loaned *Lagenophora* specimens from P was destroyed at the Australian border before they could be examined by the borrowing botanists. These specimens were examined by means of scans and are marked with a † symbol. Some nomenclatural typifications were made subsequent to this event, including that of *L. sublyrata*, discussed here (Bean and Wang 2017; Bean and Jabbour 2018). Morphological descriptions and terminology follow Harris and Harris (2001). In addition to dried specimens, live specimens were collected by the first two authors and Christian Laudereau and subsequently cultivated at the nursery of Institut Agronomique néo-Calédonien in Païta, Port-Laguerre, to make finer measurements and facilitate observations. As much as possible, localities recorded on older specimens were visited to recollect the plants and to assess the ecology where they were found. Most measurements are based on live cultivated material under binocular Olympus SZ2-ILST equipped with a camera. When dried material was used, the dimensions are based on material (i.e. florets) reconstituted with boiling water. Specimen locations were mapped with QGIS 3.10 (QGIS Development Team 2020) to generate the distribution maps and to help for IUCN (2019) evaluation assessments. The occurrence data have been uploaded to the Global Biodiversity Information Facility (**GBIF**) via the Pensoft Data Hosting Center at the GBIF’s Integrated Publishing Toolkit (**IPT**) (https://doi.org/10.15468/zkjcfx).

## Taxonomic treatment

### Key to the New Caledonia species of *Lagenophora*:

**Table:** 

1	Trichomes on scape patent, 0.2–0.5 mm long. Cypsela with beak 0.1–0.3 mm long; oval in cross section, longitudinal ribs on both surfaces, base with no trichome	***L. sinuosa***
–	Trichomes on scape appressed, c. 0.1 mm long. Cypsela with a beak 0.4–0.5 mm long; laterally flattened, smooth on both surfaces, base usually with 1–3 trichomes more or less caducous	***L. sublyrata***

#### Lagenophora sinuosa

Taxon classificationPlantaeAsteralesAsteraceae

Lannuzel, Gâteblé & Jian Wang ter sp. nov.

CF3BD18E-BF93-5364-BEE5-398DCDB0D1E9

urn:lsid:ipni.org:names:77217119-1

Figs 1 , 2

##### Type.

**New Caledonia**. **North Prov.**: Pouembout, Ouaté, 500 m, 21°9'52.43"S, 165°7'1.5"E, 26 Mar 2019, G. *Gâteblé, S. Liede-Schumann, U. Meve &D. Fleurot 1091* (***Holotype***: P!, ***isotype***: NOU107482!).

##### Diagnosis.

*Lagenophora sinuosa* Lannuzel, Gâteblé & Jian Wang ter differs from all other species in the genus with its usually deeply lobed leaf margins and ribbed cypsela surface. It resembles *L. queenslandica* Jian Wang ter & A.R.Bean with its very short cypsela beak.

##### Description.

***Perennial rhizomatous herb*;** roots and rhizomes fibrous; stem usually absent (leaves in basal rosette); leaves and scapes firmly attached to stem and/or rootstock. ***Leaves*** 5–10, oblanceolate to spathulate, 1.5–4 cm long by 0.5–1.5 cm wide (1.5–3 × longer than wide), winged petiole-like base 0.1–3 cm long; leaf apex obtuse to rounded; leaf margins more or less deeply lobate, sinuate to crenate, usually with 4–10 deep lobes, each lobe 2–5 mm deep; upper leaf surface greyish green; with 4–10 trichomes per mm^2^, each 0.3–1 mm long; lower leaf surface pale green, with 1–6 trichomes per mm^2^, each 0.2–0.6 mm long; leaf margins with 6–14 trichomes per mm, each 0.7–1 mm long; secondary veins obscure on upper leaf surface, but sometimes obvious on lower leaf surface. ***Scapes*** channelled or not, 1–4 per tuft, 3–25 cm long, c. 0.5 mm diameter; bracts 2–7, upper ones c. 4 × 0.5 mm, lower ones c. 5.0 × 1.5 mm; trichomes 0.2–0.5 mm long, patent or retrorse, erect; 10–15 trichomes per mm^2^ at midpoint of scape, 15–30 trichomes per mm^2^ towards apex; papillae to c. 0.01 mm long, 5–15 per mm^2^ at midpoint of scape, but very densely distributed towards apex. ***Capitula*** 3–6 mm long, 2–5 mm diameter; involucral bracts c. 25 in 2–3 rows, with trichomes c. 0.3 mm occasionally along midrib on outer surface, linear to lanceolate, apex purple, acute to acuminate, with fringed margins on distal half, outer bracts 1.3–2.1 × 0.5 mm, inner bracts 2–3.5 × 0.3–0.7 mm. ***Receptacle*** convex, 2.8–4.2 mm diameter and 1.4–2.5 mm high. ***Ray florets*** 20–30 in 1 or 2 rows; tube 0.1–1 mm long, c. 0.3 mm wide, glandular pilose; style branches c. 0.5 mm long; ligules 1.9–4.2 × 0.4–0.9 mm, with longitudinal veins obscure, white or very occasionally pink, apex obtuse and bidentate. ***Disc florets*** 10–30, corolla greenish or yellow, tubular, 1.5–2 mm long, outer surface covered with papillae; corolla lobes 4–5, deltate, 0.4–0.6 × 0.3 mm; stamens 4–5, anthers 0.6–0.8 mm long; style branches 0.4–0.9 mm long; sterile ovary 2.2–2.5 mm long; pappus scales absent. ***Cypselae*** oval in cross section, oblanceolate, 2.3–3.2 × 0.5–0.8 mm excluding beak, uniformly brown at maturity; surfaces with 2–4 longitudinal ribs on each side; no trichome at the base; beak 0.1–0.3 mm long, densely covered by glands, without a thickened white annular collar at its apex.

**Figure 1.**
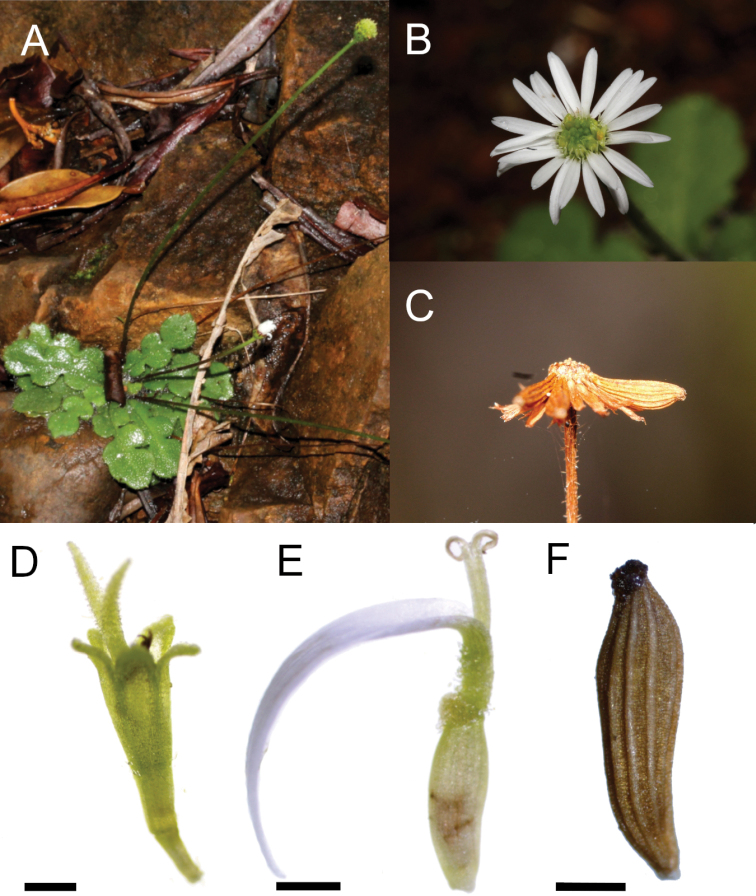
*Lagenophora sinuosa* Lannuzel, Gâteblé & Jian Wang ter, sp. nov. **A** plant in natural conditions **B** capitula **C** capitula with mature cypsela **D** disc floret **E** ray floret **F** cypsela **A–D** from *Gâteblé 1184***E, F** from *Gâteblé 1187*. Photos from G. Lannuzel. Scale bars: 0.5 mm.

##### Additional specimens examined.

**New Caledonia. North Prov.**: Haute rivière de Voh, 250 m, 12 Mar 1951, *Guillaumin & Baumann-Bodenheim 12112* (P03292561image!†); Haute rivière de Voh, 250 m, 12 Mar 1951, *Guillaumin & Baumann-Bodenheim 12154* (P03292559image!†); Vallée de la Moindah (branche nord), 150 m, 3 Oct 1965, *MacKee 13515* (P04427664image!†); Mt Paéoua, contrefort nord-est, 600–900 m, 4 Jul 1967, *MacKee 17004* (P04427667image!†); Pouembout, 30 m, 26 May 1971, *MacKee 23675* (NOU054762!, P04234038image!); Pouembout, 30 m, 16 Feb 1972, *MacKee 25003* (P03276832image!); Pouembout, 30 m, 16 Apr 1981, *MacKee 38956* (P04427671image!†); Poya, forêt de Nékoro, 2 m, 26 May 1983, *MacKee 41502* (CANB718870.1image!, NOU054761!, P04427669image!†); Poya, forêt de Nékoro, 2 m, 16 Aug 1984, *MacKee 42136* (NOU072073!, P04295155image!†); Poya nord, entre le creek Hervouet et son affluent nord au dessus de la RT1, 40–50 m, 14 Oct 1998, *Veillon 8135* (NOU072074!); Cap Devert, 1861–1867, *Vieillard 816 (Deplanche? 109)* (P03292531image!†). **South Prov.**: Mont Dore, 800 ft., 3 Apr 1914, *Compton 675* (BM013867015image !); Tontouta, 1 Nov 1924, *Däniker 414* (P03292449image!†); Prony, 2 m, 22°19'31.5"S, 166°49'34.44"E, 17 Mar 2020, *Gâteblé, Lannuzel & Ititiaty 1184* (NOU107485!, P!); Cap N’Doua, Kô Mwâ Nirê, 20 m, 22°22'33.78"S, 166°56'28.1"E, 17 Mar 2020, *Gâteblé, Lannuzel & Ititiaty1187* (BRI!, K!, MPU!, NOU107484!, P!); Prony, Îlot Casy, 5 m, 22°21'20.84"S, 166°50'46.77"E, 14 Aug 2020, *Gâteblé 1224* (BRI!, K!, MPU!, NOU107486!, P!); Prony, Sep 1910, *Godefroy s.n*. (P03292558image!, left plant); Ouipouin, 21°41'18.96"S, 165°59'25.08"E, 14 Dec 2018, *Laudereau 1236* (NOU091404!); Sommet de la Table Unio (1000 m), 21 Sep 1965, *MacKee 13414* (P04427666image!†); Plateau sommital de la Table Unio, 1000 m, 14 Nov 1970, *MacKee 22908* (NOU072308!, NSW935348, P04427668image!†); Mont Nakada, 1000 m, 21°37'50"S, 166°3'35"E, 18 Apr 2001, *Munzinger & McPherson 814* (P00217314image!); Ouaménie, 1 Jul 2006, *Munzinger et al. 3488* (NOU013890!); Poya, sud-est de Mépouiri, 10 m, 9 Jul 1991, *Veillon 7390* (NOU072081!); Colline à M’bée, 1855–1860, *Vieillard 816* (P03292447image!†).

##### Probable additional specimen.

Haute Tipindje, Contrefort Sud du massif Oua Tilou, 400 m, 28 Jun 1970, *MacKee 22128* (P03292503image!†). We were not able to conclusively identify this specimen as the image apparently bears no cypsela. Regarding morphology and ecology, it may be a *L. sinuosa* but further field work is needed to acquire certitude on that locality.

##### Distribution and habitat.

**Figure 2.**
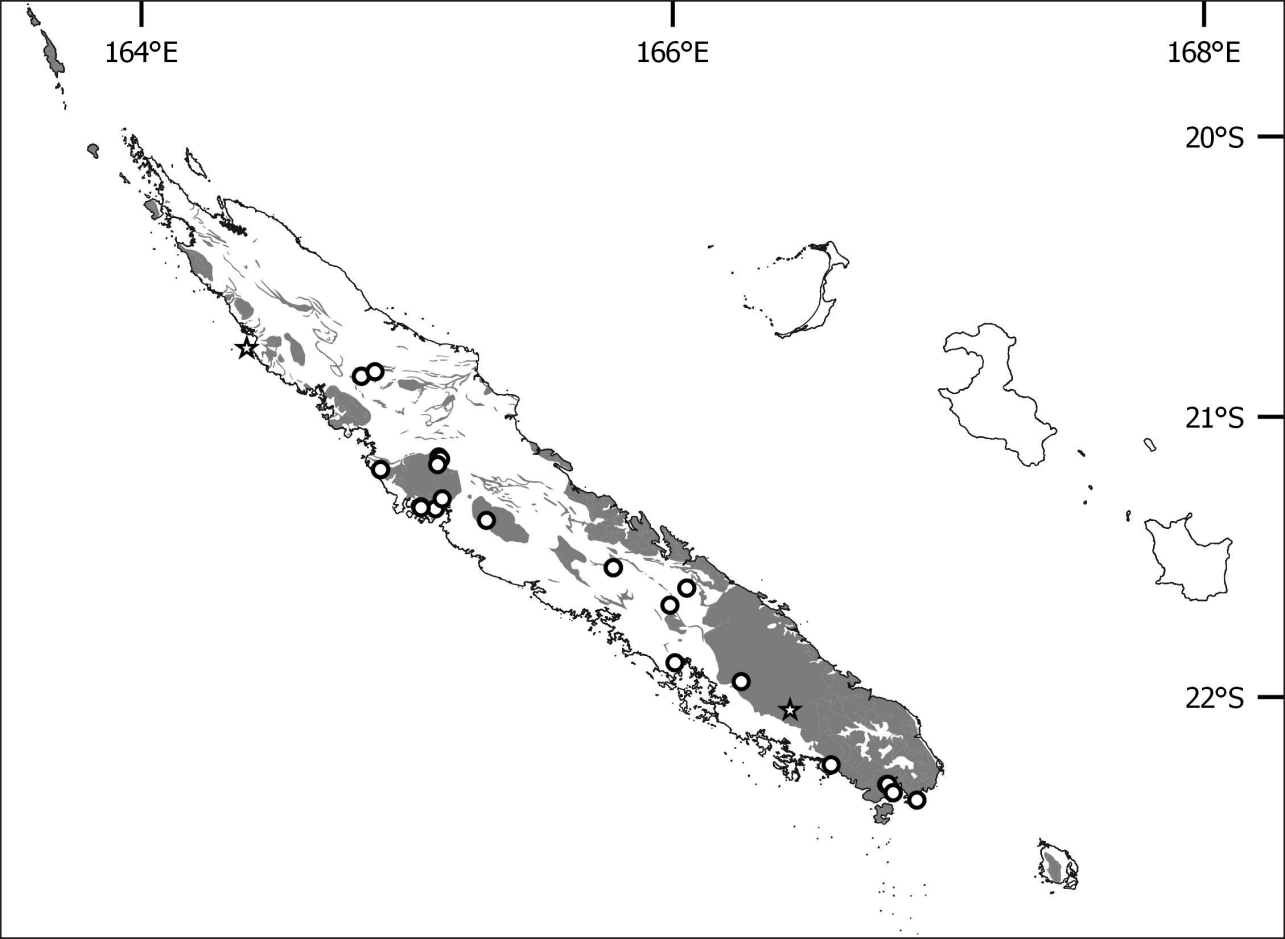
Distribution of *Lagenophora sinuosa* Lannuzel, Gâteblé & Jian Wang ter, sp. nov. Dots represent reliable localities and stars doubtful ones, grey shaded areas represent ultramafic outcrops.

*Lagenophora sinuosa* is an endemic species to New Caledonia. It grows only on mainland Grande Terre from the southern tip to Kaala-Gomen as the northernmost locality. As with *L. sublyrata*, the species has a relatively broad altitudinal distribution ranging from 2 to 1000 m above sea level (Fig. 2). It inhabits the wet forests and open scrublands (maquis minier), mainly on serpentinic alluvium but has been recorded also on peridotitic derived soils at higher altitude and on vertisols at low altitudes.

##### Phenology.

Both flowers and fruits were recorded from February through November from herbarium specimens.

##### Etymology.

The specific epithet *sinuosa* refers to the sinuate leaf margins, by which the species differs from *L. sublyrata*. Some immature plants or populations bear crenate leaves in natural conditions, but showed ability to produce deeply lobed leaves in greenhouse conditions.

##### Conservation status.

The species is largely distributed on the mainland, though often neglected by collectors, perhaps because it is an inconspicuous herb and maybe considered as an “exotic” or weedy species. The number of localities where it occurs may then be underestimated through herbarium records. The ecology of the species being rainforests floors and maquis on both ultramafic and non-ultramafic substrates at low to medium altitudes tend to consider the invasive introduced Rusa deer (*Rusa timorensis*) as the major threat both by grazing and by trampling. The fire threat is another issue, especially for open maquis populations. Nevertheless, with over ten localities (sensu IUCN 2019) recorded, *L. sinuosa* does not meet the requirements for a threatened species and qualifies for the Least Concern (LC) status.

##### Notes.

*Lagenophora sinuosa* has variable leaf shapes. Although its leaf margins are usually deeply lobed, there are two populations growing in the understory of coastal *Araucaria* forests showing crenate leaf margins. These two populations were considered at one stage as a different species. However, further examinations of their fertile aspects showed there were no significant differences between these two and all other populations. Moreover, some individuals of two of the populations that were cultivated in greenhouse conditions with fertilizers can occasionally produce lobed leaves. Therefore, these two populations have been included in *L. sinuosa*.

Generic placement of the new species is subject to debate, and consideration was given to making it a new monotypic genus. However, as pointed out by Saldivia et al. (2020), rank redundancy with monotypic genera is already relatively high (36%) in Asteraceae. Thus, considering existing genera in the region, this new species is here included in the genus *Lagenophora* because of the involucres with 1 to 2–seriate ray florets, the disc florets with 4–5 corolla lobes, and cypselae with a glandular beak. The new species is atypical in the genus because of its distinctive but variable leaf shapes and more importantly its cypselae that have longitudinal ribs on both surfaces. Its characteristics were also compared with other genera in the subtribe *Lagenophorinae*, including the closely related genera in the region, viz. *Pytinicarpa* G.L.Nesom, *Solenogyne* Cass., *Keysseria* Lauterb. and *Myriactis* Less., or the morphologically similar *Brachyscome* Cass. in Brachyscominae. In Table 1 also a comparison with other members of the genus is made. Further molecular based studies among these related genera, and including New Caledonian samples, are highly recommended to clarify its position.

**Table 1.** Comparison of the diagnostic characters of *Lagenophora sinuosa* and related genera with data modified from Hind (2004).

Taxon	Marginal florets	Disc florets	Cypselae	Pappus
*Lagenophora sinuosa*	1–2–seriate with ligule	4–or 5–lobed, functionally male	ribbed, with glandular beak	absent
*Lagenophora* (other species)	2–5–seriate with ligule	4– or 5–lobed, functionally male	smooth, with glandular beak	absent (except scales on disc florets of *L. sublyrata*)
* Brachyscome *	uniseriate with ligule	4– or 5–lobed, fertile or sterile	ribbed or not, no glandular beak	present
* Keysseria *	2– or 3–seriate with ligule	4–lobed, sterile	smooth, with glandular beak	absent
* Myriactis *	2–5(–10) –seriate with ligule	4– or 5–lobed, fertile or sterile	smooth, with glandular beak	absent
* Pytinicarpa *	uniseriate with ligule	5–lobed, sterile	ribbed, no glandular beak	present (barbellate)
* Solenogyne *	3– or 4–seriate without ligule	4–lobed, functionally male	smooth, no glandular beak	absent

#### Lagenophora sublyrata

Taxon classificationPlantaeAsteralesAsteraceae

(Cass.) A.R.Bean & Jian Wang ter, Austrobaileya 10: 435. 2019.

7EB0D58B-C0B4-5DBC-AC6B-0B7F9FA81E6B

Figs 3 , 4

Ixauchenus sublyratus Cass. in F.Cuvier, Dict. Sci. Nat. 2^nd^ ed. 56: 176. 1828. Type: New South Wales. Port Jackson, Nov.-Dec. 1819, *C. Gaudichaud* (Lectotype: P 00742955, image only extant, fide Bean and Wang (2017).
Ixauchenus lyratus Less., Syn. Gen. Compos. 193. 1832, *nomen nudum*.
Lagenophora billardierei var. media DC., Prodr. 5: 307. 1836. Type: Nova Hollandia, 1823, *F.W. Sieber 505* (Syntypes: G 00454010!, HAL, NY 00180436!).
Lagenophora billardierei var. glabrata DC., Prodr. 5: 307. 1836. Type: Nova Hollandia, without locality, 1816, from Lambert’s herbarium (Syntype: G 00454009!).
Lagenophora lanata A.Cunn., Ann. Nat. Hist. 2: 126. 1838. Type: New Zealand. Between the Waitangy and Keri-Keri Rivers, 1834, *R. Cunningham 437* (Lectotype: K000890104!, *fide*Allen 1961: 606).

##### Type.

**Australia. New South Wales**: Port Jackson, November–December 1819, *C. Gaudichaud* (***Lectotype***: P 00742955†, image only extant; designated by Bean and Wang 2017); **Australia. New South Wales**: Hornsby, April 1914, *W.F. Blakely s.n.* (***Epitype***: NSW 10275!, designated by Bean and Wang 2017).

##### Description.

***Perennial rhizomatous herb***; roots fleshy, 0.2–1 mm diameter; no obvious stem; leaves and scapes firmly attached to rootstock. ***Leaves*** 4–9(–11), obovate, oblanceolate, elliptical or spathulate, 1–6 cm long by 0.6–1.6 cm wide (c. 2.5 ×longer than wide), sessile or with a winged petiole-like base to 1 cm long; leaf apex obtuse; leaf margins toothed, crenate to sinuate, with 2–10 teeth, each tooth c. 1 mm long; upper leaf surface green, with 2–7 trichomes per mm^2^, each 0.3–0.6 mm long; lower leaf surface pale green, with 3–7 trichomes per mm^2^, each 0.1–0.4 mm long; leaf margins with 6–12 trichomes per mm, each 0.1–0.4 mm long; net veins usually obscure on dried material on both surfaces. ***Scapes*** channelled or not, 1–6 per tuft, 5–11(–22) cm long, 0.5–0.8 mm diameter; bracts 2–4, upper ones c. 1.1 × 0.2 mm, lower ones 1.1–2.9 × 0.4 mm; trichomes c. 0.1 mm long, antrorse, more or less appressed; 10–30 trichomes per mm^2^ at midpoint of scape, slightly denser towards apex. ***Capitula*** 3.1–3.5 mm long, 1.8–4 mm diameter; involucral bracts 15–20 in 2–3 rows, glabrous, lanceolate, oblong to obovate, apex green or purple, obtuse to acute, ciliate on distal part, outer bracts 1.3–1.9 × 0.6 mm, inner bracts 2.1–2.7 × 0.7–0.8 mm. ***Receptacle*** convex, 0.6–0.8 mm diameter and 0.5–0.8 mm high. ***Ray florets*** 20–30 in 2 rows; tube c. 0.4 mm long, 0.1–0.2 mm wide, glandular pilose; style branches c. 0.4 mm long; ligules 1.8–2.6 mm × 0.5–0.6 mm, with obscure longitudinal veins, white, creamy, or purple with age, apex obtuse or bidentate. ***Disc florets*** 6–11, corolla light yellow, tubular, 1.5–1.8 mm long, outer surface with sparse glandular trichomes; corolla lobes 5, deltate, 0.2–0.3 × 0.4–0.5 mm; stamens 5, anthers c. 0.6 mm long; style branches 0.3–0.6 mm long; sterile ovary 0.6–0.7 mm long; pappus scales 1 or 2, c. 0.1 mm long. ***Cypselae*** laterally compressed, lanceolate or obliquely oblanceolate, 2.2–2.4 × 0.4–0.8 mm excluding beak, light brown to dark brown at maturity; cypsela edges more or less thickened, smooth; with 1–3 eglandular more or less caducous trichomes present usually at base of cypsela; cypsela glands confined to dorsal side of beak and adjacent area of cypsela; cypsela beak 0.4–0.5 mm long, with a thickened white annular collar at its apex, 0.15–0.2 mm diameter.

**Figure 3.**
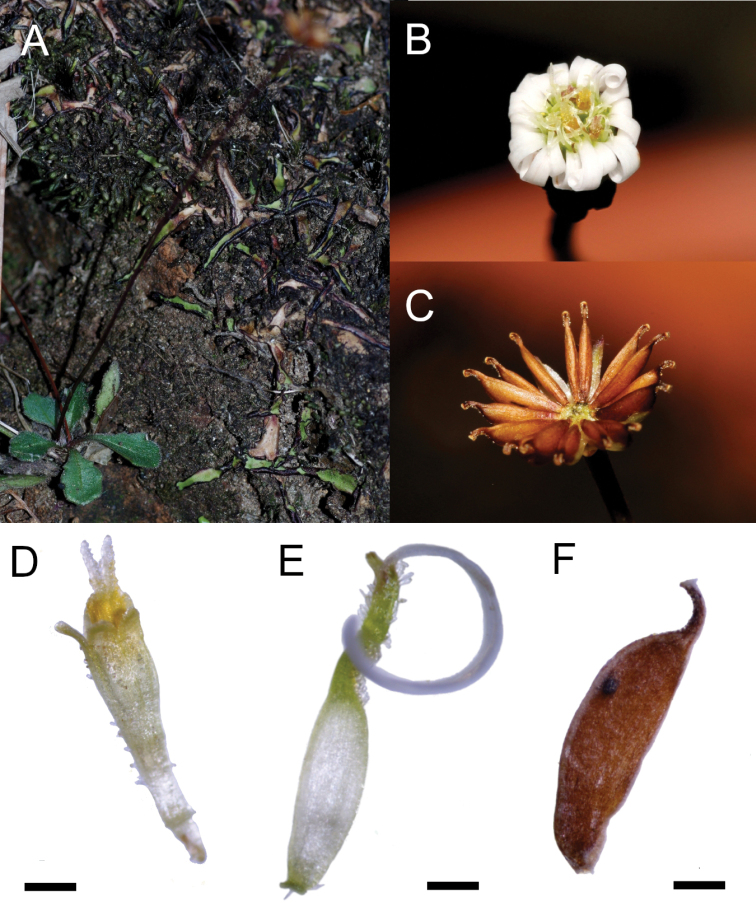
*Lagenophora sublyrata* (Cass.) A.R.Bean & Jian Wang ter. **A** plant in habitat **B** capitula **C** capitula with mature cypsela **D** disc floret **E** ray floret **F** cypsela **A–F** from *Lannuzel 348*. Photos from G. Lannuzel. Scale bars: 0.5 mm.

##### Specimens examined.

**New Caledonia. North Prov.**: Mont Mi, 9 Mar 1869, *Balansa 1023* (P03292499image!†); Col des Roussettes, 537 m, 15 Sep 1964, *Blanchon 963* (NOU072076!); Ouaté, 21°9'19.98"S, 165°9'6.98"E, 15 Apr 2019, *Laudereau 1268* (NOU091405!); Diahoué, 20°28'53.11"S, 164°41'33.29"E, 28 Jul 2019, *Laudereau 1286* (NOU091406!); Mt Pouitchate between upper Tipindjé and upper Kamendoua above Ateu, 1000 m, 29 Aug 1956, *MacKee 5139* (L1815294image!, P03292522image!†); Contrefort de la roche Ouaième, 400 m, 27 Dec 1964, *MacKee 11865* (P03292502image!†); Haute Diahot, forêt de Tendé, exploitation forestière Frouin, 500 m, 31 Mar 1969, *MacKee 20470* (NOU072078!, P03276833image!, P04234036image!); Tiwaka, Moindip, 550 m, 31 Mar 1974, *MacKee 28455* (P04427679image!†); Col Maré, Amoa-Tiwaka, 500 m, 13 Aug 1977, *MacKee 33612* (CANB718871.1!, NOU072079!, P04427665image!†); Pouébo, Ouangati, 700 m, 20 Oct 1978, *MacKee (legit Cherrier)35947* (P04427672image!†); Néhoué, vallée de la Rade, 50 m, 8 Mar 1979, *MacKee 36698* (NOU072077!, P04427673image!†); Piémont sud du Kantalupaik, 300 m, 20°51'6.012"S, 165°0'36"E, 1 Nov 2017, *Pignal, Munzinger & Bruy 5263* (P01073109image!); Région de Pouembout au nord de Forêt Plate, 25 Mar 1981, *Suprin 1079* (NOU072080!); Plateau de Tango, 650 m, 20 Oct 1981, *Veillon 4555* (NOU072082!, P04427674image!†); Cap Tonnerre, 1861–1867, *Vieillard 816* (P03292525image!†); sur la montagne à Balade, 1855–1860, *Vieillard 817* (P03292446image!†, P03292497image!†); Gatope, 1861–1867, *Vieillard 817* (P03292496image!†). **South Prov.**: Dumbéa, *Baudouin 498* (P03292450image!†); Mont Mou summit, 3500 ft, 15 Mar 1914, *Compton 574* (BM013867014image!, P03292498image!†); Mts Koghis, 300 m, 25 Jan 1927, *Franc 486* (P03292501image!†); 1874–1876, *Germain s.n*.(P03292500image!†); Mé Aoui, 500 m, 8 Feb 1951, *Guillaumin & Baumann-Bodenheim 10444* (P03292560image!†); Cultivated plant at IAC Port-Laguerre, 11 Jun 2020, originally collected at Cascade de Dogny, 915 m, 21°37'04.2"S, 165°53'18.8"E, 21 Jan 2019, *Lannuzel 348* (NOU107487!); Cultivated plant at IAC Port-Laguerre, 5 Jan 2021, originally collected at Monts Koghis, sur le chemin du Pic Malaoui, 670m, 22°10'52.2"S, 166°30'43.2"E, 15 May 2020, *Lannuzel 427* (NOU107488!); Ouipouin, 21°41'17.88"S, 165°59'13.56"E, 13 Dec 2018, *Laudereau 1235* (NOU091403!); Vallée de la Thy, 400 m, 7 Jan 1956, *MacKee 3741* (L1815296image!, P03292451image!†, P03292523image!†); Slope of Mt Koghi toward Vallée de la Thy (St Louis), 400–500 m, 24 May 1956, *MacKee 4651* (L1815293image!, P03292520image!†); Col d’Amieu, Mont Pembai, 600 m, 15 Apr 1976, *MacKee 31018* (NSW935344!, P04427663image!†); Dogny, la cascade, 26 Oct 2007, *Munzinger 4621* (NOU030729!); Monts Koghis, propriété Lavoix, 11 Mar 1966, *Nothis 67* (NOU072075!); Ile des Pins, 1860s, *Pancher 473* (P03292495image!†); Auf den Hügeln bei Yaouhé, 150 m, 25 Sep 1902, *Schlechter 14804* (L1815295image!, P03292494image!†).

##### Probable additional specimen.

Some plants on *Vieillard 817*, sur la montagne à Balade, 1855–1860 (P03292448image!†), see notes.

##### Distribution and habitat.

**Figure 4.**
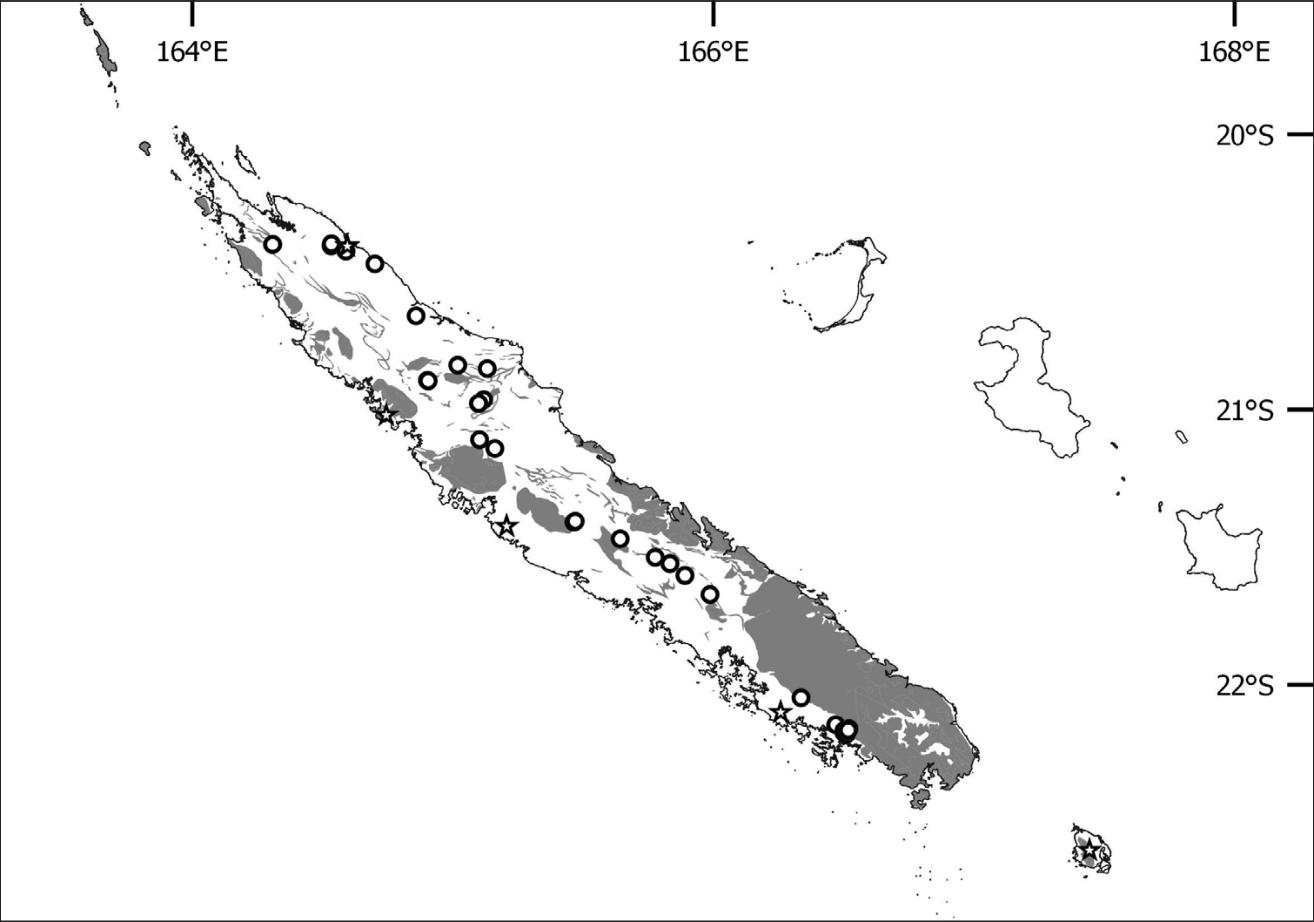
Distribution of *Lagenophora sublyrata* in New Caledonia. Dots represent reliable localities and stars doubtful ones, greyed areas represent ultramafic outcrops.

*Lagenophora sublyrata* is the most widespread species in the genus ranging from India and Sri Lanka to south-east Asia (e.g. Vietnam), China, Taiwan, Japan, Indonesia (e.g. Java), New Guinea, Australia and New Zealand. In New Caledonia, it has a widespread distribution (Fig. 4) on mainland Grande-Terre with only one old specimen on the outer island of Île des Pins (if the *Pancher* locality is correct). It mainly inhabits the wet grounds of rainforests or rainforests margins though some specimens are collected in more open conditions (e.g. *Franc 486*, *Lannuzel 348*, *MacKee 33612*& *35947*). The species seems to avoid soils derived from ultramafic substrate, except for *Compton 574* which was recorded on ultramafic outcrop (Fig. 4) and is recorded from 50 m to 1200 m above sea level but is usually growing at medium altitudes (400–600 m a.s.l.).

##### Phenology.

Flowers and fruits have been recorded almost all year round with a peak of specimens in March but this could be an artefact. In cultivation, the species seems to flower throughout the year.

##### Conservation status.

The species is relatively common on mainland, though often neglected by collectors, perhaps because it is inconspicuous or considered to be an exotic or weedy species. Hence, the number of localities where it occurs, judging by herbarium records, may be underestimated. The ecology of the species is rainforest on non-ultramafic substrates at low to medium altitudes. The invasive-introduced Rusa deer (*Rusa timorensis*) may represent a major threat through overgrazing or by trampling of herbaceous vegetation. Nonetheless, with over ten localities (sensu IUCN 2019) recorded, *L. sublyrata* does not meet the requirements for a threatened species and qualifies for the Least Concern (LC) status.

##### Notes.

*Lagenophora sublyrata* is a widespread species with variable leaf shape, indument and plant size. New Caledonia specimens are usually smaller in stature than typical plants from eastern Australia, but features of the roots, cypselae, scapes and involucral bracts are consistent with it. The specimen *MacKee11865* bears two numbers on it; 11864 on the Paris herbarium label and on the wrapper and 11865 on a manuscript label by MacKee himself. MacKee’s field notebook (held at NOU herbarium) shows that 11864 is a *Mitrasacme* Labill., and 11865 is a *Lagenophora* sp. The correct collection number is therefore 11865. The specimen *Vieillard 817* (P03292448image!†) is a mixed specimen with plants of both *L. sublyrata* and *L. sinuosa*. Vieillard used a confusing system of numbering of herbarium specimens and mixed specimens are well-known (see Hopkins and Bradford 2009; Morat 2010). An additional problem is the mounting of several plants on the same sheet, probably done by Sébastien-René Lenormand at Institut Botanique de Caen (CN) before the New Caledonian collection was sent to P.

## Supplementary Material

XML Treatment for Lagenophora sinuosa

XML Treatment for Lagenophora sublyrata
